# News Items

**Published:** 1868-04-15

**Authors:** 


### *EDITORIAL.*

---

***News Items.***

Prof. E. S. Carr has resigned his position in the University of Wisconsin.——It is said a new Medical College is being organized in Detroit, Michigan, to offset, probably, the Ann Arbor *fiasco*.——H. H. Childs, M.D., long connected with Berkshire Medical College, is dead.——The Legislature of Wisconsin, at the instance of Dr. D. C. Davies, of Portage City, has passed a liberal law legalizing dissections. Also, to prohibit quacks from giving testimony in courts on medical matters, and from collecting fees. Other stringent legislation, at our last advices, was contemplated. Dr. D. is an energetic worker, and will make his mark in the State of his choice. ——Rush Medical College appears to have had the largest graduating class at its recent commencement as yet reported. The facilities for medical instruction and observation in this city can scarcely be surpassed, at the present time, in any Atlantic city. When the dog in the manger stops howling, there will be no difficulty in rivaling the proudest cities of the old world. God speed the day, even though it result in a Governor or Congressman.——Our friend, D. C. Warner,

M.D., has established a first class Pharmacy and Dispensing Drug Store, on the corner of Indiana Avenue and Twenty-Third Street. This will be good news to the denizens of that rapidly growing division of Chicago. The Doctor can be relied on "every time," either as physician, druggist or dentist. (This latter is the editorial soliloquy.)

